# Compression of the Inferior Vena Cava in Bowel Obstruction

**DOI:** 10.1155/2013/469297

**Published:** 2013-09-14

**Authors:** Alessandro Cina, Roberto Zamparelli, Sara Venturino, Riccardo Gargaruti, Vittorio Semeraro, Franco Cavaliere

**Affiliations:** ^1^Department of Bioimaging and Radiological Sciences, Catholic University of the Sacred Heart, 00168 Rome, Italy; ^2^Institute of Anaesthesia and Intensive Care, Catholic University of the Sacred Heart, 00168 Rome, Italy

## Abstract

*Introduction*. We investigated whether (a) the inferior vena cava (IVC) is compressed in bowel obstruction and (b) some tracts are more compressed than others. *Methods*. Two groups of abdominal computed tomography (CT) examinations were collected retrospectively. Group O (*N* = 69) scans were positive for bowel obstruction, group C (*N* = 50) scans were negative for diseases. IVC anteroposterior and lateral diameters (APD, LAD) were assessed at seven levels. *Results*. In group C, IVC section had an elliptic shape (APD/LAD: .76 ± .14), the area of which increased gradually from 1.9 (confluence of the iliac veins) to 3.1 cm^2^/m^2^ of BSA (confluence of the hepatic veins) with a significant narrowing in the hepatic section. In group O, bowel obstruction caused a compression of IVC (APD/LAD: .54 ± .17). Along its course, IVC section area increased from 1.3 to 2.5 cm^2^/m^2^. At ROC curve analysis, an APD/LAD ratio lower than 0.63 above the confluence of the iliac veins discriminated between O and C groups with sensitivity of 74% and specificity of 96%. *Conclusions*. Bowel obstruction caused a compression of IVC, which involved its entire course except for the terminal section. APD/LAD ratio may be useful to monitor the degree of compression.

## 1. Introduction

Abdominal compartment syndrome (ACS) is a life-threatening condition in which organ failure originates from the increase of intra-abdominal pressure (IAP) [1–6]. Intra-abdominal hypertension (IAH) impairs cardiovascular, hepatic, renal, and gastrointestinal functions, mainly by compressing abdominal blood vessels and neurologic and pulmonary functions by shifting the diaphragm upwards. In critically-ill patients, ACS is associated with a significant increase of mortality [7]. Early recognition of IAH is critical for an effective ACS treatment, but is hampered by the poor sensitivity of clinical examination [8]. Bladder and gastric pressure measurements remain the cornerstone to diagnose and monitor IAH [9], but borderline values may be misinterpreted and should be evaluated cautiously because patient size and position and the application of positive end-expiratory pressure affect measurements [10–12]. Furthermore, mild IAP elevations may be clinically significant only in the presence of hypovolemia [13] or arterial hypotension [14]. 

Imaging can be of value in patients affected by IAH or ACS. Computed tomography (CT) and ultrasounds (US) of the abdomen may show mosaic liver perfusion, bowel or gastric wall enhancement, diaphragm elevation, and an increased ratio between anteroposterior and transverse abdominal diameters [15, 16]. Besides, echocardiography can effectively assess the hemodynamic status [17] and Doppler ultrasounds may unveil changes of renal vascular resistances, which are well correlated with IAH severity [18, 19]. Sonographic assessment of abdominal vein size and flow has been recently carried out during IAH simulated by a corset tightly enveloped around the abdomens of healthy volunteers [20]. In such a condition, the compression of the inferior vena cava (IVC) assessed at the confluence of renal veins was constantly present and easily quantified by calculating the ratio between anteroposterior and lateral diameters. This finding suggested that IVC compression may hypothetically provide a useful tool to monitor early hemodynamic effects of IAH.

Bowel dilation secondary to a mechanical obstruction occupies an increasing intraperitoneal volume and compresses other structures. When compensatory mechanisms are exceeded, an increase of IAP occurs [21], which may result in ACS development [22]. The aim of this study was to verify whether patients affected by intestinal obstruction undergo a compression of IVC similar to that observed in simulated IAH and if such a compression affects parts of IVC other than the confluence of renal veins. A secondary purpose was to assess whether such changes are more pronounced in some segments of the vein so that sonographic assessment at patient bedside may be more sensitive at those levels. 

## 2. Materials and Methods

From the picture archiving and communication system (PACS) and radiology information system (RIS) of the Catholic University hospital in Rome, starting from October 2010 and going back through the previous months, we collected 119 consecutive computed tomography examinations (CT) of the abdomen, which had been performed in order to (a) substantiate the clinical diagnosis of mechanical intestinal obstruction and in which the diagnosis was positively supported by the presence of diffuse or localized distension of the bowel associated with air-fluid levels (group O, *N* = 69) or (b) investigate the presence of secondary localizations of neoplasms previously removed with surgery and in which no lesion was observed (group C, *N* = 50). CT collection was carried out backwards until at least 50 cases were gathered in both groups. Criteria of exclusion were, (a) patient age lower than 18 years, (b) the presence of lesions that could directly affect the size of IVC by compression or attraction, and (c) examinations done with technical modalities different from those described here. The study received the approval of the institutional Ethics Committee.

All the CT examinations were performed with Lightspeed 16 or Highspeed 64 scanners (General Electric, Wakeshua, IL, USA), using 2.5 mm-thick slices and acquisition starting 80 seconds after IV administration of 120 mL of nonionic iodated contrast medium (350 mg/dL). All CT images were evaluated on professional workstations (Advantage Windows 4.3, General Electric, Wakeshua, IL, USA) by the same radiologist. Two groups of measures were carried out as follows.

**Figure 1:**
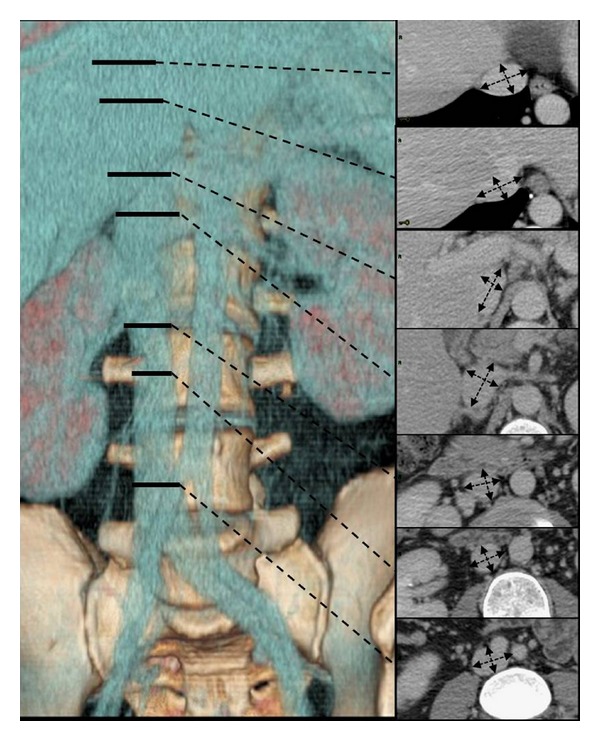
Levels at which IVC was evaluated. The seven levels at which IVC was evaluated are reported on a 3D Volume Rendering reconstruction and the corresponding axial images are shown. APD and LAD were measured by taking into account the rotation of the vena. Levels: (1) 1 cm after the confluence of the iliac veins, (2) 1 cm before the confluence of the renal veins, (3) just before the confluence of the renal veins, (4) just after the confluence of the renal veins, (5) 1 cm after the confluence of the renal veins, (6) just before the confluence of the supra-hepatic veins, and (7) just after the confluence of the supra-hepatic veins.

- IVC antero-posterior and lateral diameters (APD, LAD, resp.) were assessed at the following levels (Figure 1): (1) 1 cm above the confluence of the iliac veins, (2) 1 cm below the confluence of the lower renal vein, (3) just at the confluence of the lower renal vein, (4) just at the confluence of the higher renal vein, (5) 1 cm above the confluence of the higher renal vein, (6) just below the confluence of the hepatic veins, and (7) just above the confluence of the hepatic veins. The diameters were measured by taking into account IVC obliquity [23], so that antero-posterior and lateral axes coincided with the major and minor axes of the elliptic section of the vein (Figure 1). At each level, the IVC section area was calculated with the formula for the area of an ellipse (*π*  ∗ LAD/2 ∗ APD/2).
- In order to evaluate patient body surface, the first lumbar vertebra, or L1, was located and the centralmost section through L1 was selected. At that level, the transverse diameter of L1 (L1TD), the area of L1 (L1A), the spinal canal area (SCA), and the body transverse diameter (BTD) and circumference (BC) were measured. The body surface area (BSA) was then calculated according to the following formulas [24].
- Men:
   BSA = −0.615293 + (0.041699 × L1A) + (0.088085 × SCA) − (0.006687 × age) +(0.231837 × BTD).
- Women:
   BSA = −0.724995 + (0.019472 × BC)−(0.003817 × age)+(0.062063 × L1TD).

In 19 CTs included in group O, IAP values measured in the four hours before the execution of the abdominal CT were recorded. IAP was determined with a standard procedure, by measuring vesicle pressure after injecting 20 mL of saline with the patient in the horizontal position and the pressure transducer in correspondence of the mid-axillary line. 

### 2.1. Statistical Analysis

Data are reported as means (standard deviations). Statistical analysis was performed by utilizing the software “Statistica 6” (StatSoft, USA). Comparisons between groups about sex, age, and anthropometric parameters were performed with chi-square test and *t*-test for independent samples. IVC diameters were analyzed by three-way (groups/levels/APD Vs LAD) ANOVA for repeated measures; APD/LAD ratios and IVC section areas were analyzed with two-way (groups/levels) ANOVA for repeated measures. Post-hoc comparisons were performed with Tukey's test. Receiver operating characteristic (ROC) analysis was employed to assess the optimal cut-off for APD/LAD ratio in order to differentiate patients with intestinal obstruction from controls. ROC curve analysis was performed with the software MedCalc version 9.2.1.0 (MedCalc Software, B). Significance was assumed for *P* values < 0.05.

## 3. Results

Table 1 reports patient age, sex, and BSA; no significant difference was observed between the two groups. In the group O, all patients were hospitalized in surgical wards because their clinical conditions did not require the admission to an intensive care unit; all examinations were performed within 48 hours of hospitalization. Bowel obstruction was situated in the jejunum (*N* = 24), the ileum (*N* = 17), the right and descending colon (*N* = 15), or the sigmoid colon and the rectum (*N* = 13). The abdominal pressure value correspondent to 19 TC examination in group O was 12.9 (3.7) mmHg; abdominal hypertension defined as abdominal pressure higher than 11 mmHg [4] was present in 12 cases (63%).

**Figure 2:**
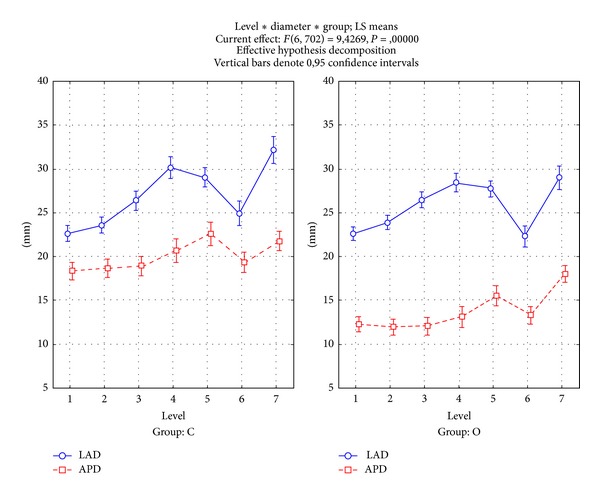
IVC diameters in groups O (bowel obstruction) and C (controls). Antero-posterior (AP, continuous lines) and lateral (LA, dotted lines) diameters in groups O (bowel obstruction) and C (controls) at levels (1)–(7). Vertical bars are the .95 confidence intervals of the means. ANOVA interaction among diameters, levels, and groups: *P* = .0000. Levels: (1) 1 cm after the confluence of the iliac veins, (2) 1 cm before the confluence of the renal veins, (3) just before the confluence of the renal veins, (4) just after the confluence of the renal veins, (5) 1 cm after the confluence of renal veins, (6) just before the confluence of the supra-hepatic veins, and (7) just after the confluence of the supra-hepatic veins.

Table 2 presents IVC diameters. Some findings were common to both groups of patients. First, APD was significantly shorter than LAD at all the levels investigated (*P* = .0000), so that IVC presented an elliptic section during all its abdominal course. Second, APD and LAD varied depending on the level (interaction between diameter and level, *P* = .0000), (Figure 2) is showing similar trends in the two groups. A minimum was registered at the most peripheral level evaluated, 1 cm above the confluence of the iliac veins (level 1); successively, both diameters increased until a maximum situated at the confluence of the renal veins (levels 4 and 5); when IVC entered its stretch into the liver, diameters briskly decreased until a minimum, which was observed below the confluence of the hepatic veins (level 6); and, finally, IVC size increased again to a new maximum before entering the thorax (level 7). 

The main difference between groups O and C concerned APD, which was significantly shorter in the group O (interaction diameter group, *P* = .0000). Since LAD values did not differ between the two groups (Figure 2), IVC was flatter in the group O. Imaging clearly showed that IVC was squeezed between the peritoneum and the spine or the right psoas muscle and that the degree of compression was not affected by the localization of the bowel obstruction. The degree of compression varied in relation to levels; APD was mainly 36% shorter in the group O than in the group C near the confluence of the lower renal vein (levels 2 and 3) and only 17% above the confluence of the hepatic veins (level 7). 

**Figure 3:**
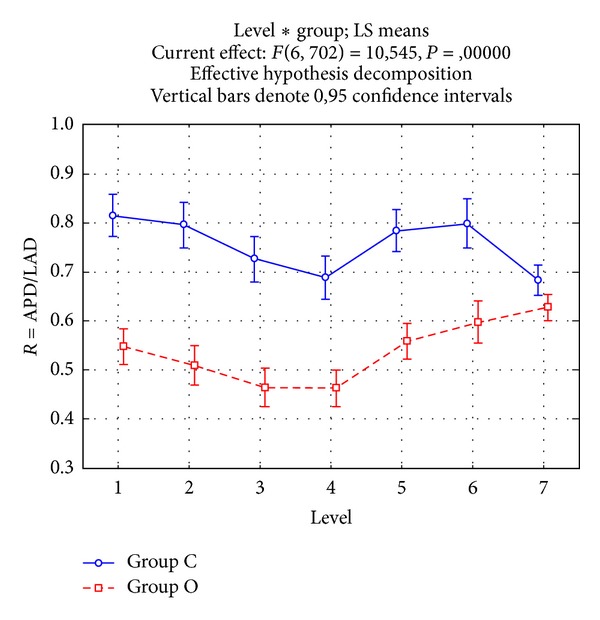
The ratio between antero-posterior and lateral diameters in groups O (bowel obstruction, continuous line) and C (controls, dotted line). Vertical bars are the .95 confidence intervals of the means. ANOVA interaction among levels and groups, *P* = .0000. Levels: (1) 1 cm after the confluence of the iliac veins, (2) 1 cm before the confluence of the renal veins, (3) just before the confluence of the renal veins, (4) just after the confluence of the renal veins, (5) 1 cm after the confluence of the renal veins, (6) just before the confluence of the supra-hepatic veins, and (7) just after the confluence of the supra-hepatic veins.

**Figure 4:**
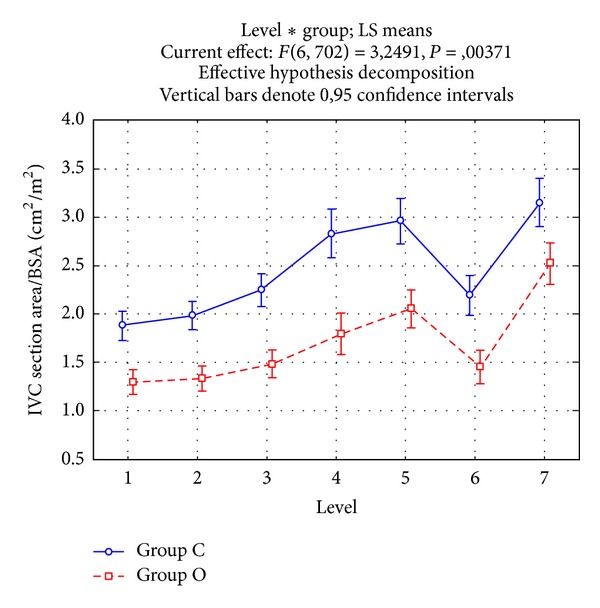
IVC section area normalized for body surface area in groups O (bowel obstruction, continuous line) and C (controls, dotted line). Vertical bars are the .95 confidence intervals of the means. ANOVA interaction among levels and groups, *P* = .0100. Levels: (1) 1 cm after the confluence of the iliac veins, (2) 1 cm before the confluence of the renal veins, (3) just before the confluence of the renal veins, (4) just after the confluence of the renal veins, (5) 1 cm after the confluence of the renal veins, (6) just before the confluence of the supra-hepatic veins, and (7) just after the confluence of the supra-hepatic veins.

The APD/LAD ratio expresses the eccentricity of IVC elliptic section, the lower the ratio, the flatter the vein at that level. In both groups, the lowest ratio was observed at the confluence of the renal veins, but values were significantly lower in the group O than in the group C (*P* = .0000) (Figure 3). Instead, at the confluence of the hepatic veins, the two groups presented similar values. The IVC section area varied along the course of the vein mirroring APD and LAD changes (Figure 4). In both groups, the area increased progressively, apart from a narrowing that occurred in the intrahepatic part. The area was significantly smaller in the group O than in the group C at all levels, apart from level 7. 

**Figure 5:**
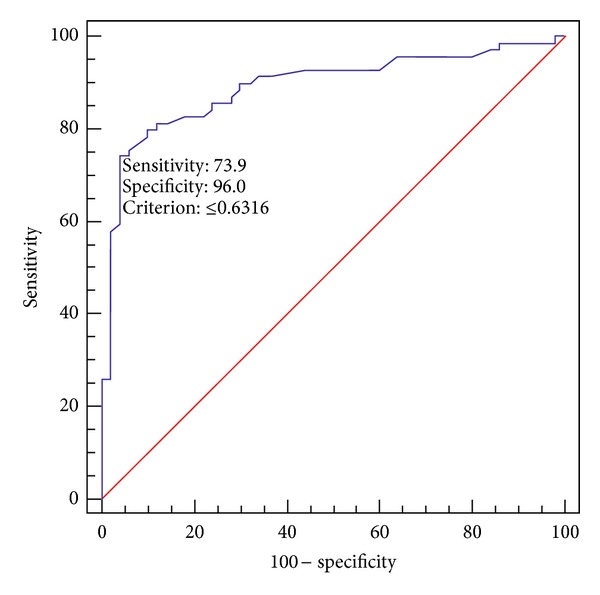
ROC curve of the ratio between anteroposterior and lateral caval diameters 1 cm above the iliac vein confluence for differentiating patients with abdominal obstruction from controls. Ten steps of ratio from 0.10 to 1.00. Cut-off value = 0.63; this value was associated with 74% sensitivity and 96% specificity.

At ROC analysis, an APD/LAD ratio of 0.63 measured 1 cm above the confluence of the iliac veins was the optimal cut-off for differentiating controls from patients with abdominal obstruction; this value was associated with 74% sensitivity and 96% specificity (Figure 5).

## 4. Discussion

Our study points out that in bowel obstruction, likewise in simulated IAH, IVC undergoes a compression against the psoas muscle and the spine. The compression is along the entire abdominal course of the vein. Consequently, IVCs already elliptic shape becomes more eccentric and the section area decreases by about 30%, which may potentially impair blood flow and ultimately blood return to the right ventricle. IVC is not compressed uniformly along its course, but the initial part up to the confluence of the renal veins is more affected than the hepatic section; the last part, just before entering the thorax, is the least compressed. Consequently, ultrasound at patient bedside should focalize on the first part of IVC to detect the compression by the dilated bowel.

IVC is a compressible conduct, the size and shape of which are affected by the balance between the external pressure, which depends on IAP, and the internal pressure. In group O, the complete IVC collapse observed by other authors [15, 25] never occurred, probably because the compression from the outside was balanced by the increase of the intraluminal pressure due to the higher resistance to blood flow. 

In group O, IVC was deformed throughout the entire abdominal course, but compression was less pronounced in the hepatic tract and near the diaphragm. Data collected by the US in patients affected by IAH were not univocal on this point. Some authors reported an increase of IVC caliber at the confluence of the hepatic veins, others a decrease [26]. According to our data, in patients affected by bowel obstruction, the caliber becomes larger at this level and APD/LAD ratio and section area are not significantly different from controls. These findings suggest that the last portion of IVC is less compressed because it is protected by the liver. As IVC is more compressed in its initial portion, sonographic measurements to monitor IVC compression in bowel obstruction could be better performed in this portion (up to the confluence of the lower renal vein) rather than in the following sections. On the other hand, our data suggest that the final portion of IVC is particularly suitable to estimate central venous pressure because it is less affected by compression from outside. Finally, the IVC lateral diameter did not change in patients affected by bowel obstruction, possibly because retroperitoneal pressure did not increase. Alternatively, IVC size may be scarcely influenced by retroperitoneal pressure. During laparoscopic surgery, the IVC internal pressure increased during peritoneal insufflations, but did not change during retroperitoneal ones [27].

Both the section area and APD/LAD ratio may be utilized to monitor the degree of IVC compression in patients affected by bowel obstruction. In simulated IAH, a section area lower than one cm^2^/m^2^ discriminated IAH from the baseline condition [20]. However, in the present study, we observed many section areas larger than one cm^2^/m^2^ both in patients affected by bowel obstruction and in controls. This inconsistency may be explained because CT images were taken in the supine position at the end of a forced inspiration, whereas in the previous study US measurements were performed in the Morrison position at the end of expiration. Nonetheless, the influence of patient position and of the respiratory cycle may reduce the usefulness of the IVC section area to quantify IVC compression. We, therefore, tested the accuracy of the APD/LAD ratio to discriminate group O from group C. We found that a value of 0.66 provided a good level of sensitivity and specificity. On this purpose, IVC diameters are better measured in the infrarenal IVC, where compression is more pronounced. 

Bowel dilation secondary to a mechanical obstruction can cause an increase of IAP [21], but IAH develops only when compensatory mechanisms are overcome. In this study, the IAP values registered in correspondence to 19 CTs included in group O, showed that most patients had mild IAH, but about one third did not. Consequently, IVC compression was strictly associated with bowel obstruction, but did not necessarily indicate the presence of IAH. This finding suggests that IVC compression may be a very early sign during the course of bowel obstruction, when compensatory mechanisms are still effective in limiting IAP increase. Further studies are needed to verify the existence of a relationship between the degree of IVC compression and IAP values.

Finally, our study provides some data on the IVC normal size and shape. A progressive increase of size, which was more pronounced at the confluence of the renal veins, was observed as far as sections approximated the diaphragm. That trend was interrupted by a significant narrowing (27%) in the hepatic portion of the vein. At that level, pathological narrowing can occur in liver cirrhosis, in which IVC can be compressed by an enlarged caudate lobe [28], and in IAH [29]. A pressure gradient occurring in the hepatic section of IVC may have positive effects. Increased upstream pressure may counterbalance IAP, while decreased downstream pressure may favor venous drainage through the hepatic veins. 

## 5. Conclusion

Patients affected by bowel obstruction present a compression of IVC against the spine and the psoas muscle, which is clearly shown by CT scan with the application of contrast medium, but may be effectively assessed with ultrasounds at patient's bedside. Albeit with the limitation of the relatively small number of cases analyzed, our data suggest that the APD/LAD ratio may well quantify the degree of compression and be an additional diagnostic tool for detecting IAH. On this purpose, the ratio should be assessed in the initial part of the vein up to the confluence of the lower renal vein because compression is more pronounced at that level. 

## Figures and Tables

**Table 1:** Age, sex, and body surface area (BSA) in groups O (bowel obstruction) and C (controls).

	Group O	Group C	*P *
*N*	69	50	
Males/females	29/40	24/26	n.s.
Age	67.9 (18.2)	63.3 (15.6)	n.s.
BSA	1.71 (.25)	1.77 (.24)	n.s.

**Table 2:** Anteroposterior (APD) and lateral (LAD) IVC diameters. (1) 1 cm after the confluence of the iliac veins, (2) 1 cm before the confluence of the renal veins, (3) just before the confluence of the renal veins, (4) just after the confluence of the renal veins, (5) 1 cm after the confluence of the renal veins, (6) just before the confluence of the supra-hepatic veins, and (7) just after the confluence of the supra-hepatic veins. Data are reported as means (standard deviations). In group O, significant differences against the correspondent value in group C at Tukey's test are distinguished by the use of bold characters.

	1	2	3	4	5	6	7
Group O (obstruction)							
APD mm	**12.2** **(4.1)**	**12.0** **(4.3)**	**12.0** **(4.6)**	** 13.1** **(5.2) **	**15.5** **(5.2) **	**13.3** **(4.4)**	18.0 (4.4)
LAD mm	22.6 (3.6)	23.9 (3.8)	26.5 (4.1)	28.4 (5.2)	27.7 (4.2)	22.3 (4.7)	29.0 (6.0)
APD/LAD	**.55** **(.18)**	**.51** **(.19)**	**.46** **(.19)**	**.46** **(.17)**	**.56** **(.17)**	**.60** **(.16)**	.63 (.13)
AREA cm^2^/m^2^	**1.30** **(.54)**	1.33 (.55)	**1.48** **(.63)**	**1.79** **(.92)**	2.06 (.86)	1.45 (.75)	2.52 (1.02)

Group C (control)							
APD mm	18.4 (2.9)	18.7 (3.0)	18.9 (3.1)	20.7 (4.3)	22.6 (3.8)	19.3 (3.9)	21.8 (3.0)
LAD mm	22.6 (2.8)	23.6 (2.4)	26.4 (3.3)	30.1 (3.2)	29.0 (3.4)	24.9 (5.2)	32.2 (4.6)
APD/LAD	.82 (.11)	.80 (.13)	.73 (.14)	.69 (.14)	.78 (.13)	.80 (.21)	.68 (.09)
AREA cm^2^/m^2^	1.88 (.51)	1.98 (.47)	2.25 (.58)	2.83 (.86)	2.96 (.77)	2.19 (.72)	3.15 (.72)

## Abbreviations

ACS: Abdominal compartment syndrome
APD: Inferior vena cava antero-posterior diameter
BSA: Body surface area
CT: Computed tomography
IAH: Intra-abdominal hypertension
IAP: Intra-abdominal pressure
IVC: Inferior vena cava
LAD: Inferior vena cava lateral diameter
ROC: Receiver operating characteristic
US: Ultrasounds.
